# Supplementary material for: Biomarkers of Vascular Injury and Type 2 Diabetes: A Prospective Study, Systematic Review and Meta-Analysis
Source: J Clin Med. 2019 Nov 27;8(12):2075. doi: 10.3390/jcm8122075 (PMC6947572; doi:10.3390/jcm8122075)
Supplement: Supplementary file 1 [file jcm-08-02075-s001.pdf]

Search Strings

PubMed search string (MEDLINE)

((((("e-selectin" OR “sE-selectin” OR "E Selectin" OR "endothelial dysfunction" OR “p-selectin” OR “sP-Selectin” OR “ICAM3” OR “fibrinogen” OR “glycoprotein IIb/IIIa” OR “thrombomodulin” or “thrombopoietin”) AND (diabet*) AND (epidemiology OR cohort OR prospective OR "population-based" OR “follow-up” OR longitudinal)))))

Web of Science search string

TS = ((((("e-selectin" OR “sE-selectin” OR "E Selectin" OR "endothelial dysfunction" OR “p-selectin” OR “sP-Selectin” OR “ICAM3” OR “fibrinogen” OR “glycoprotein IIb/IIIa” OR “thrombomodulin” or “thrombopoietin”) AND (diabet*) AND (epidemiology OR cohort OR prospective OR "population-based" OR “follow-up” OR longitudinal)))))

Covariates Assessment

Information on covariates was obtained at the baseline study examinations (1994-1998), through a detailed medical interview (including questionnaire assessments of physical activity, smoking and alcohol intake as well as education level), anthropometric measurements, and biomarkers from blood samples. Hypertension was defined as patient declared diagnosis, systolic blood pressure ≥140, diastolic blood pressure ≥90 (both available only for 50% of the study population), or use of antihypertensive medication (anatomical therapeutic chemical classification system C02, C03, C04, C05, C07, C08, and C09). Serum samples were sent on dry ice to Scandinavian Health Ltd. laboratories (Etten-Leur, Netherlands) for basic clinical chemistry measurements, including serum concentrations of CRP, total cholesterol, HDL-cholesterol, and tryglicerides. All measurements were made using the Roche Cobas 6000 analytical system for clinical chemistry (Roche Diagnostics, Mannheim, Germany), according to the manufacturer’s protocols. LDL-cholesterol (mmol/l) was calculated based on the Friedewald formula ($\mathrm{LDL} = \text{total cholesterol} - \mathrm{HDL} - (\text{triglycerides}/5)$) [1].

Statistical Analyses

Simple conversion of an effect estimated between quantiles of a continuous exposure to a 1-standard deviation change in exposure. Assuming the underlying continuous exposure to follow a normal distribution, exposure quantiles can be represented by their expected values, derived as expected value of a standard normal distribution truncated at corresponding quantile limits. Because one unit in the standard normal distribution is equivalent to one standard deviation (std), the difference (delta) between the quantiles’ expected values represents a change in terms of std. Thus an effect estimated between quantiles *βquantilediff,* may be regarded as an effect between the quantiles’ expected values and can be converted to the corresponding effect of 1 std change in the continuous exposure as $\beta_{1std} = \frac{\beta_{quantilediff}}{delta_{quantiles}}$, where $delta_{quantiles} = E(upper\_quantile) - E(lower\_quantile)$.

For a truncated standard normal distribution with probability density function φ and cumulative density function Φ the expected value within a quantile of length *quantilelength* can be derived as

$$E(quantile) = \frac{\varphi(lowerlimit) - \varphi(upperlimit)}{\Phi(upperlimit) - \Phi(lowerlimit)} = \frac{\varphi(lowerlimit) - \varphi(upperlimit)}{quantilelength},$$

and *lowerlimit* and *upperlimit* can be derived from the inversed cumulative density function Φinv of the lower and upper percentile limits of the quantiles in perspective.

For example: converting an effect estimated between top and bottom quartile, for the lower quartile we derive $E(1st\ quartile) = \frac{0 - \varphi(\Phi^{inv}(0.25))}{0.25} = \frac{-\varphi(-0.674)}{0.25} = -1.27$, and correspondingly $E(4th\ quartile) = \frac{\varphi(\Phi^{inv}(0.75)) - 0}{0.25} = \frac{\varphi(0.674)}{0.25} = 1.27$, which gives $delta_{quartiles\ 4\ to\ 1} = 1.27 - (-1.27) = 2.54$ and so $\beta_{1std} = \frac{\beta_{quartile4\ vs\ 1}}{2.54}$.

List of the difference (delta) between the quantiles' expected values deltas for comparison of tertiles, quartiles, and quintiles.

Quantile	Top vs. bottom	Delta
Tertile	2 vs. 1	1.09
	3 vs. 1	2.18
Quartile	2 vs 1	0.95
	3 vs 1	1.60
	4 vs 1	2.54
Quintile	2 vs 1	0.87
	3 vs 1	1.40
	4 vs 1	1.93
	5 vs 1	2.80

Supplementary Table 1. Newcastle-Ottawa Quality Assessment (self-adjusted).

Assessment of quality of a cohort study – Newcastle Ottawa Scale		
Selection (tick one box in each section)		
1.	Representativeness of the exposed cohort	
a)	truly representative of the average, elderly, community-dwelling resident ★	☐
b)	somewhat representative of the average, elderly, community-dwelling resident ★	☐
c)	selected group of patients, e.g. only certain socio-economic groups/areas	☐
d)	no description of the derivation of the cohort	☐
2.	Selection of the non-exposed cohort	
a)	drawn from the same community as the exposed cohort ★	☐
b)	drawn from a different source	☐
c)	no description of the derivation of the non-exposed cohort	☐
3.	Ascertainment of exposure	
a)	secure record (eg health care record) ★	☐
b)	structured interview ★	☐
c)	written self-report	☐
d)	other / no description	☐
4.	Demonstration that outcome of interest was not present at start of study	
a)	yes ★	☐

b)	no	☐
Comparability (tick one or both boxes, as appropriate)		
1.	Comparability of cohorts on the basis of the design or analysis	
a)	study controls for age, sex ★	☐
b)	study controls for any additional factors (BMI, smoking) ★	☐
Outcome (tick one box in each section)		
1.	Assessment of outcome	☐
a)	independent blind assessment ★	☐
b)	record linkage ★	☐
c)	self-report	☐
d)	other / no description	
2.	Was follow up long enough for outcomes to occur	
a)	yes, if median duration of follow-up ≥ 5 years ★	☐
b)	no, if median duration of follow-up < 5 years	☐
3.	Adequacy of follow up of cohorts	☐
a)	complete follow up: all subjects accounted for ★	☐
b)	subjects lost to follow up unlikely to introduce bias: number lost <= 20%,★ or description of those lost suggesting no different from those followed	☐
c)	follow up rate < 80% (select an adequate %) and no description of those lost	☐
d)	no statement	

Supplementary Table 2. Newcastle-Ottawa Quality Assessment for each study

Study / Items	Selection				Comparability	Outcome			Total
	**1**	**2**	**3**	**4**	**1**	**1**	**2**	**3**	
Duncan, 1999[2]	1	1	1	1	2	1	1	0	8
Festa, 2002[3]	1	1	1	1	2	1	1	0	8
Krakoff, 2003 [4]	1	1	1	1	1	1	1	0	7
Meigs, 2004[5]	1	1	1	1	2	1	1	0	8
Thorand, 2006[6]	1	1	1	1	2	1	1	1	9
Song, 2007 [7]	1	1	1	1	2	1	1	0	8
Thorand, 2007[8]	1	1	1	1	2	1	1	0	8
Stranges, 2008[9]	1	1	1	1	2	1	1	0	8
Bertoni, 2010[10]	1	1	1	1	2	1	0	0	7
Dallmeier, 2012[11]	1	1	1	1	2	1	1	0	8
Julia, 2014 [12]	1	1	1	1	2	1	1	0	8
Odegaard, 2016[13]	1	1	1	1	2	1	1	0	8
Pankow, 2016[14]	1	1	1	1	2	1	1	0	8
De Simone, 2017[15]	1	1	1	1	2	1	0	0	7
Pletsch-Borba, 2019	1	1	1	1	2	1	1	1	9

Numbers refer to the number of stars given to each criterion.

Supplementary Table 3. Missing data in the EPIC-Heidelberg subcohort (n=2224 participants)

	T2D cases (n = 163)	Non-cases (n = 2,061)	Overall (n = 2,224)
Age at recruitment (years)	0(0%)	0(0%)	0(0%)
Women	0(0%)	0(0%)	0(0%)
Hypertension (yes)	0(0%)	0(0%)	0(0%)
BMI (kg/m^2)	0(0%)	0(0%)	0(0%)
Height (cm)	0(0%)	0(0%)	0(0%)
Weight (kg)	0(0%)	0(0%)	0(0%)
Waist circumference (cm)	0(0%)	0(0%)	0(0%)
Alcohol intake at Baseline (g/day)	0(0%)	0(0%)	0(0%)
Education level	0(0%)	0(0%)	0(0%)
Smoking Status	0(0%)	5(0%)	5(0%)
Aspirin use (yes)	0(0%)	0(0%)	0(0%)
Antithrombotic drug use (yes)	0(0%)	0(0%)	0(0%)
Physical Activity(Cambridge index)	0(0%)	0(0%)	0(0%)
CRP (mg/l)	3(2%)	15(1%)	18(1%)
LDL (mmol/l)	23(14%)	139(7%)	162(7%)
Triglycerides (mmol/l)	23(14%)	135(7%)	158(7%)
HDL(mmol/l)	2(1%)	12(1%)	14(1%)
Total Cholesterol (mmol/l)	23(14%)	131(6%)	154(7%)
HbA_{1c}(mmol/mol)	21(13%)	152(7%)	173(8%)
E-Selectin (ng/ml)	1(1%)	2(0%)	3(0%)
P-Selectin (ng/ml)	1(1%)	1(0%)	2(0%)
ICAM3 (ng/ml)	1(1%)	1(0%)	2(0%)
Thrombomodulin (ng/ml)	1(1%)	2(0%)	3(0%)
Thombopoietin (pg/ml)	0(0%)	1(0%)	1(0%)
Glycoprotein IIb/IIIa (ng/ml)	1(1%)	20(1%)	21(1%)
Fibrinogen (μg/ml)	0(0%)	1(0%)	1(0%)

Data presented as n (%) for categorical variables. CRP indicated C-reactive protein, LDL low-density lipoprotein, HDL high-density lipoprotein, HbA_{1c} glycated haemoglobin, and ICAM3 intercellular adhesion molecule 3.

Supplementary Table 4. Within- and between-batch coefficients of variation, and intra-individual correlation coefficients across vascular injury biomarkers.

Biomarker	Within-batch coefficient of variation	Between-batch coefficient of variation *	One-year intra-individual Spearman's correlation coefficient (ϱ) **
E-Selectin	3.6%	10.6%	0.88
P-Selectin	3.3%	9.1%	0.80
ICAM3	7.5%	10.2%	0.69
Thrombomodulin	3.8%	10.1%	0.63
Thrombopoietin	4.6%	19.5%	0.73
GP IIb/IIIa	5.5%	46.9%	0.51

*For analyses on disease risks, samples of cases and non-cases were randomly assigned to analytical batches to avoid differential misclassification. Thus, between-batch variation could be addressed by statistical batch-standardization. **Derived from a subsample of $n = 78$ [16, 17]

Supplementary Table 5. Median concentrations of each biomarker in tertile of biomarkers in women and men from the EPIC-Heidelberg subcohort.

Biomarkers	Women (*n* = 1,217)			Men (*n* = 1,007)		
	Tertile 1	Tertile 2	Tertile 3	Tertile 1	Tertile 2	Tertile 3
E-Selectin (ng/ml)	5.6(4.4, 6.7)	9.12(8.2, 10.4)	13.6(22.9, 16.3)	6.9(5.7, 8.2)	10.8(9.4, 12.2)	16.1(14.1, 18.5)
P-Selectin (ng/ml)	18.3(15.4, 21.2)	25.2(23.1, 28.0)	34.6(30.8, 39.4)	21.8(18.4, 24.7)	29.8(27.0, 33.1)	40.7(35.9, 45.7)
Thrombomodulin (ng/ml)	2.2(1.9, 2.4)	2.7(2.5, 3.0)	3.4(3.1, 3.8)	2.5(2.3, 2.8)	3.0(2.8, 3.3)	3.8(3.4, 4.2)
Thrombopoietin (pg/ml)	274(237, 310)	344(315, 387)	438(390, 505)	270(236, 310)	327(297, 366)	423(373, 473)
ICAM3 (ng/ml)	0.33(0.27, 0.38)	0.44(0.38, 3.51)	0.55(0.47, 0.67)	0.36(0.30, 0.41)	0.46(0.39, 0.52)	0.61(0.52, 0.73)
GP IIb/IIIa (ng/ml)	311(273, 413)	360(324, 489)	464(393, 626)	309(267, 411)	364(322, 490)	435(381, 612)
Fibrinogen (µg/ml)	3231 (2991, 3459)	3734 (3540, 3953)	4421 (4123, 4765)	3238 (2991, 3454)	3698 (3537, 3916)	4341 (4099, 4773)

Medians (percentile25, percentile75) in tertile of biomarkers concentrations, in women and men.

Supplementary Table 6. Subgroup meta-analyses on the associations between E-Selectin and T2D risk

Variable	N	HR(95%CI)	I^2(%)	P-heterogeneity
**Location**				
USA (general population)	5	1.56[1.43; 1.69]	0%	0.78
Europe	3	1.15 [1.01; 1.32]	24%	0.25
**Blood sample collection**				
Fasting	2	1.58 [1.44; 1.73]	0%	0.96
Non-fasting	7	1.23 [1.10; 1.38]	13%	0.30
**Adjustment for covariates**				
Smoking (Yes)	6	1.35 [1.13; 1.62]	74%	0.001
No	3	1.26 [1.02; 1.56]	0%	0.68
Alcohol Consumption (Yes)	5	1.33 [1.09; 1.62]	79%	<0.001
No	4	1.31 [1.08; 1.59]	0%	0.71
Physical Activity (Yes)	5	1.32 [1.07; 1.62]	79%	<0.001
No	4	1.33 [1.16; 1.52]	0%	0.75
CRP (Yes)	3	1.11 [0.94; 1.32]	13%	0.32
No	6	1.47 [1.32; 1.63]	12%	0.34
Hypertension (Yes)	3	1.21 [0.99; 1.49]	51%	0.13
No	6	1.47 [1.32; 1.63]	12%	0.34
Blood Lipids (Yes)	3	1.16 [1.00; 1.33]	24%	0.27
No	6	1.55 [1.43; 1.68]	0%	0.81
Glucose/$HBA1_c$ (Yes)	5	1.16[1.02; 1.33]	4%	0.38
No	4	1.48 [1.32; 1.66]	23%	0.27

Results derived from random effects meta-analyses.

Supplementary Figure 1 Non-linear association between log10-E-Selectin concentration (per SD) and risk of type 2 diabetes in the EPIC-Heidelberg.

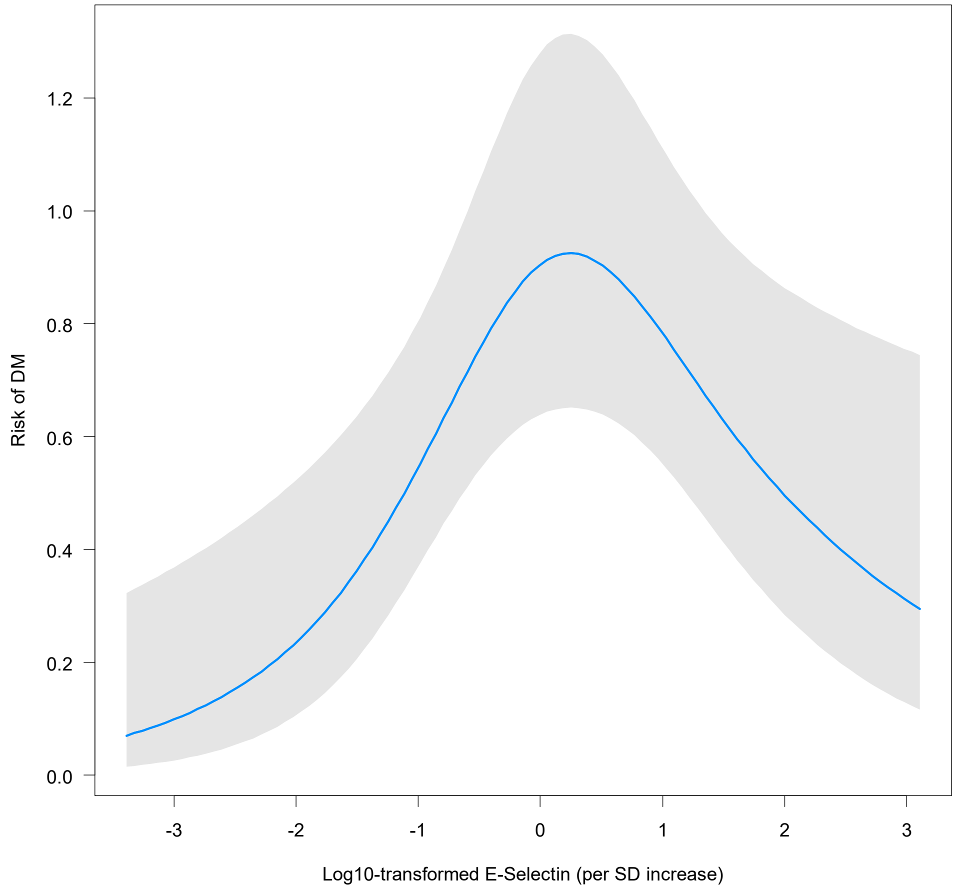

Best fitted model included natural splines with two knots. Model adjusted for age, sex, BMI (kg/m²), alcohol consumption (g/day in the past year), smoking status (never, past quitted ≥10 years ago, past quitted <10 years ago, current <15 cigarettes/day, current≥15 cigarettes/day), physical activity (Cambridge index), education level (primary, secondary and university), hypertension (yes/no), glycated haemoglobin (HbA1c) and CRP. Blue line indicates risk of diabetes per one SD increase in log10-E-Selectin concentration (batch-standardized), grey zone indicates 95% confidence interval.

Supplementary Figure 2. Meta-analysis on E-Selectin excluding one study that only showed a multivariable-adjusted model including other biomarkers of vascular injury [14].

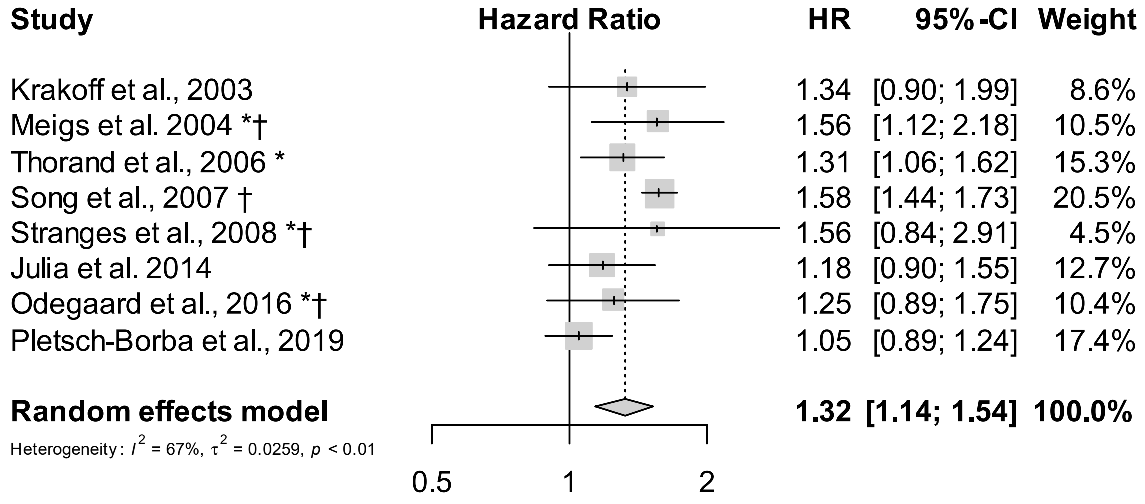

* Data derived from transformation of quantiles analyses into "per SD", † No log-transformation of the original circulating biomarker concentration performed, except for standardization (mean=0, SD=1).

Supplementary Figure 3. Meta-analysis on E-Selectin and type 2 diabetes risk excluding the present study, EPIC-Heidelberg.

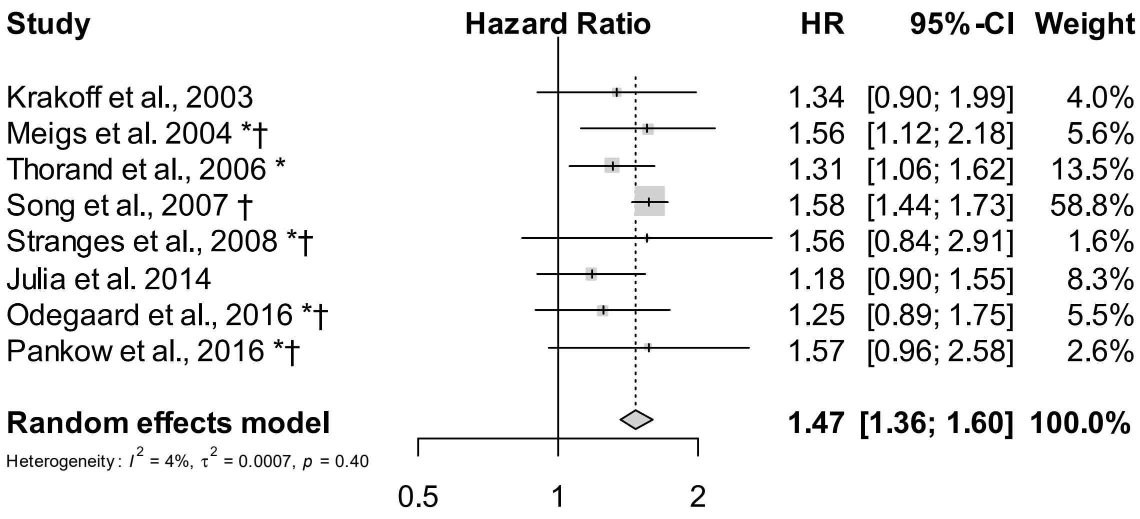

* Data derived from transformation of quantiles analyses into "per SD", † No log-transformation of the original circulating biomarker concentration performed, except for standardization (mean=0, SD=1).

Supplementary Figure 4. Meta-analysis on E-Selectin and type 2 diabetes risk excluding Thorand et al. 2006 [18] and including Herder et al. 2011 instead [19]. Both studies show results from the same study population (MONICA/KORA).

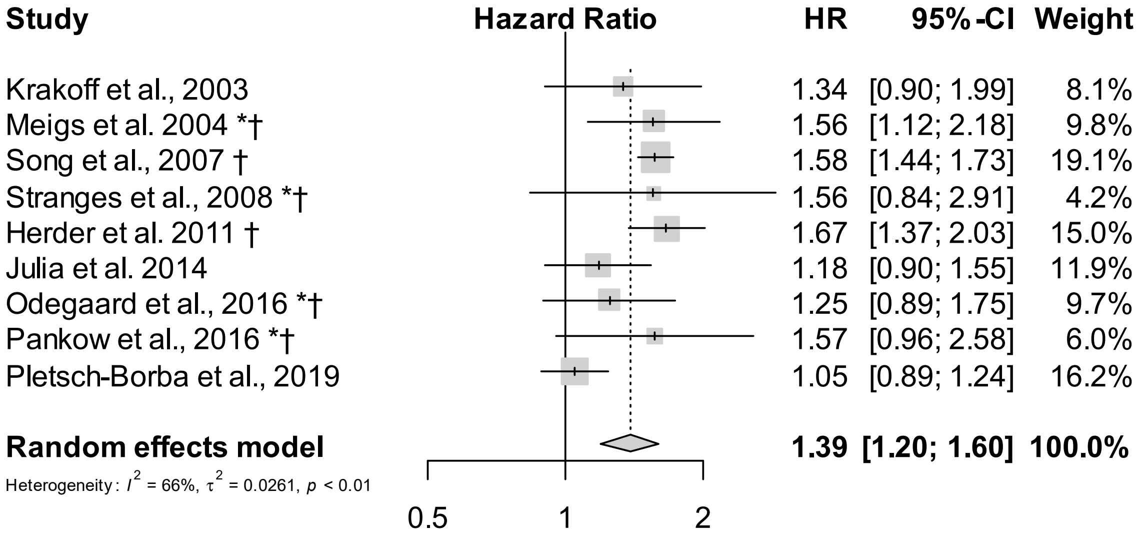

* Data derived from transformation of quantiles analyses into "per SD", † No log-transformation of the original circulating biomarker concentration performed, except for standardization (mean=0, SD=1).

.

Supplementary Figure 5. Meta-analysis on E-Selectin and type 2 diabetes risk, using the two estimates (women and men) provided by Thorand et al. 2006 [18].

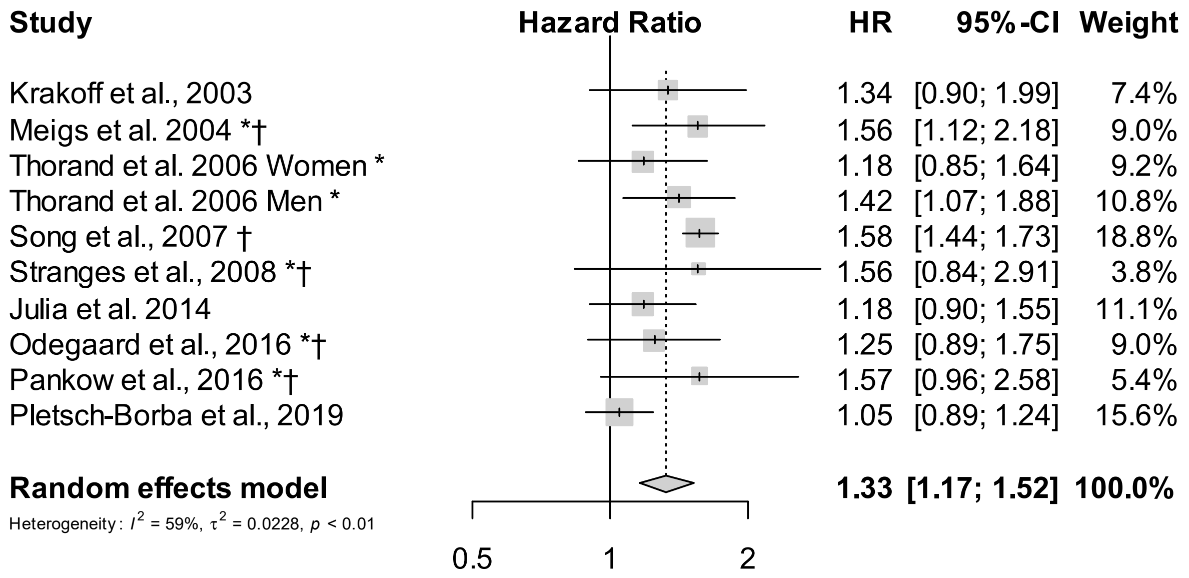

* Data derived from transformation of quantiles analyses into "per SD", † No log-transformation of the original circulating biomarker concentration performed, except for standardization (mean=0, SD=1).

References

1. Friedewald, W.T., R.I. Levy, and D.S. Fredrickson, *Estimation of the concentration of low-density lipoprotein cholesterol in plasma, without use of the preparative ultracentrifuge.* Clin Chem, 1972. **18**(6): p. 499-502.
2. Duncan, B.B., et al., *Factor VIII and other hemostasis variables are related to incident diabetes in adults - The Atherosclerosis Risk in Communities (ARIC) study.* Diabetes Care, 1999. **22**(5): p. 767-772.
3. Festa, A., et al., *Elevated levels of acute-phase proteins and plasminogen activator inhibitor-1 predict the development of type 2 diabetes - The insulin resistance atherosclerosis study.* Diabetes, 2002. **51**(4): p. 1131-1137.
4. Krakoff, J., et al., *Inflammatory markers, adiponectin, and risk of type 2 diabetes in the Pima Indian.* Diabetes Care, 2003. **26**(6): p. 1745-1751.
5. Meigs, J.B., et al., *Biomarkers of endothelial dysfunction and risk of type 2 diabetes mellitus.* JAMA, 2004. **291**(16): p. 1978-86.
6. Thorand, B., et al., *Association of cardiovascular risk factors with markers of endothelial dysfunction in middle-aged men and women. Results from the MONICA/KORA Augsburg Study.* Thromb Haemost, 2006. **95**(1): p. 134-41.
7. Song, Y., et al., *Circulating levels of endothelial adhesion molecules and risk of diabetes in an ethnically diverse cohort of women.* Diabetes, 2007. **56**(7): p. 1898-1904.
8. Thorand, B., et al., *Soluble thrombomodulin as a predictor of type 2 diabetes: results from the MONICA/KORA Augsburg case-cohort study, 1984-1998.* Diabetologia, 2007. **50**(3): p. 545-8.
9. Stranges, S., et al., *Additional contribution of emerging risk factors to the prediction of the risk of type 2 diabetes: Evidence from the Western New York Study.* Obesity, 2008. **16**(6): p. 1370-1376.
10. Bertoni, A.G., et al., *Inflammation and the incidence of type 2 diabetes: the Multi-Ethnic Study of Atherosclerosis (MESA).* Diabetes Care, 2010. **33**(4): p. 804-10.
11. Dallmeier, D., et al., *Addition of Inflammatory Biomarkers Did Not Improve Diabetes Prediction in the Community: The Framingham Heart Study.* Journal of the American Heart Association, 2012. **1**(4).
12. Julia, C., et al., *Relationships between adipokines, biomarkers of endothelial function and inflammation and risk of type 2 diabetes.* Diabetes Res Clin Pract, 2014. **105**(2): p. 231-8.
13. Odegaard, A.O., et al., *Oxidative stress, inflammation, endothelial dysfunction and incidence of type 2 diabetes.* Cardiovasc Diabetol, 2016. **15**: p. 51.
14. Pankow, J.S., et al., *Circulating cellular adhesion molecules and risk of diabetes: the Multi-Ethnic Study of Atherosclerosis (MESA).* Diabet Med, 2016. **33**(7): p. 985-91.
15. de Simone, G., et al., *Target organ damage and incident type 2 diabetes mellitus: the Strong Heart Study.* Cardiovasc Diabetol, 2017. **16**(1): p. 64.
16. Pletsch-Borba, L., et al., *Biomarkers of vascular injury in relation to myocardial infarction risk: A population-based study.* Sci Rep, 2019. **9**(1): p. 3004.
17. Graf, M.E., et al., *Biological reproducibility of circulating P-Selectin, Thrombopoietin, GPIIb/IIIa and Thrombomodulin over one year.* Clin Biochem, 2017. **50**(16-17): p. 942-946.
18. Thorand, B., et al., *Elevated markers of endothelial dysfunction predict type 2 diabetes mellitus in middle-aged men and women from the general population.* Arterioscler Thromb Vasc Biol, 2006. **26**(2): p. 398-405.
19. Herder, C., et al., *Immunological and Cardiometabolic Risk Factors in the Prediction of Type 2 Diabetes and Coronary Events: MONICA/KORA Augsburg Case-Cohort Study.* Plos One, 2011. **6**(6).
